# Automated Glioblastoma Segmentation Based on a Multiparametric Structured Unsupervised Classification

**DOI:** 10.1371/journal.pone.0125143

**Published:** 2015-05-15

**Authors:** Javier Juan-Albarracín, Elies Fuster-Garcia, José V. Manjón, Montserrat Robles, F. Aparici, L. Martí-Bonmatí, Juan M. García-Gómez

**Affiliations:** 1 Grupo de Informática Biomédica (IBIME), Instituto de Aplicaciones de las Tecnologías de la Información y de las Comunicaciones Avanzadas (ITACA), Universitat Politècnica de València, Valencia, Spain; 2 Veratech for Health S.L., Valencia, Spain; 3 Servicio de Radiología, Área imagen Clínica, Hospital Universitario y Politécnico La Fe, Valencia, Spain; 4 Department of Radiology and GIBI230 Research Group on Biomedical Imaging, Hospital Universitario y Politécnico La Fe, Valencia, Spain; Universitat de Valencia, SPAIN

## Abstract

Automatic brain tumour segmentation has become a key component for the future of brain tumour treatment. Currently, most of brain tumour segmentation approaches arise from the supervised learning standpoint, which requires a labelled training dataset from which to infer the models of the classes. The performance of these models is directly determined by the size and quality of the training corpus, whose retrieval becomes a tedious and time-consuming task. On the other hand, unsupervised approaches avoid these limitations but often do not reach comparable results than the supervised methods. In this sense, we propose an automated unsupervised method for brain tumour segmentation based on anatomical Magnetic Resonance (MR) images. Four unsupervised classification algorithms, grouped by their structured or non-structured condition, were evaluated within our pipeline. Considering the non-structured algorithms, we evaluated K-means, Fuzzy K-means and Gaussian Mixture Model (GMM), whereas as structured classification algorithms we evaluated Gaussian Hidden Markov Random Field (GHMRF). An automated postprocess based on a statistical approach supported by tissue probability maps is proposed to automatically identify the tumour classes after the segmentations. We evaluated our brain tumour segmentation method with the public BRAin Tumor Segmentation (BRATS) 2013 Test and Leaderboard datasets. Our approach based on the GMM model improves the results obtained by most of the supervised methods evaluated with the Leaderboard set and reaches the second position in the ranking. Our variant based on the GHMRF achieves the first position in the Test ranking of the unsupervised approaches and the seventh position in the general Test ranking, which confirms the method as a viable alternative for brain tumour segmentation.

## Introduction

Medical imaging techniques play a key role for brain tumour diagnosis due to the intracranial location and the unspecificity of clinical symptoms of such lesions [1]. The early identification and delineation of the different tissues related to the tumour becomes crucial to make decisions that can improve the patient survivability. The manual analysis and segmentation of these tissues involves a complex, time-consuming and biased task, which caught the attention of the Pattern Recognition (PR) and Machine Learning (ML) community [2]. Particularly, GlioBlastoma Multiforme (GBM) tumour has received most of this attention, as it is the most common and aggressive malignant tumour of the central nervous system [3, 4]. GBMs are heterogeneous lesions that present different areas of active tumour, necrosis and edema, all of them exhibiting a high variability related to the aggressiveness of the tumour. Hence, the automated segmentation of these lesions becomes a desired solution from the clinical standpoint and an interesting challenge to address from the ML community.

Recent extensive reviews of brain tumour segmentation have been presented in [2, 5]. Most of these techniques fall into the supervised learning approach. In [6, 7] Support Vector Machines (SVM) were applied to multiparametric MR datasets to segment health and pathological tissues, and additionally subcompartiments inside these areas. Jensen *et al.* [8] applied several neural networks to detect brain tumour invasion. Lee *et al.* [9] used a combination of Conditional Random Fields (CRF) and SVM to perform tumour segmentation. Bauer *et al.* [10] also used SVM and Hierarchical CRF to segment both healthy and tumour tissues including sub-compartments. Recently, Random Forest (RF) [11] techniques have shown high success in the supervised brain tumour segmentation task. In [12–15] several approaches based on variants of the RF algorithm were proposed for the Image Segmentation Challenge of Medical Image Computing and Computer-Assisted Intervention (MICCAI) 2013 Conference, reaching the first positions in the competition.

However, supervised learning requires an expensive, time-consuming and biased task to retrieve a sufficiently large set of labelled samples from which to learn discriminant functions for the posterior segmentation [5]. Furthermore, the supervised approaches are limited to the size and quality of the dataset, among other limitations such as the over-fitting to the training corpus [16]. Moreover, spatio-temporal changes in clinical environment such as new MR machines, protocols or centres may distort the data and hence could affect the performance of the supervised models [17].

Unsupervised learning tackles these limitations in a more straightforward way. Unsupervised learning does not require a training dataset from which to learn the models of the classes, but directly uses the patient specific data to find natural groupings of observations, called clusters. Hence, unsupervised learning builds an intra-patient segmentation model, which is independent from the differences between other patient’s data. By the opposite, the absence of a previous manual segmentations to guide the learning process makes the segmentation more challenging and often lead to a worse performance with respect to supervised approaches.

Some attempts for brain tissue segmentation have been made under the unsupervised paradigm. Fletcher *et al.* [18] proposed an approach based on fuzzy clustering and domain knowledge for multi-parametric non-enhancing tumour segmentation. Domain knowledge and parenchymal tissue detection is based on heuristics related to geometric shapes and locations, which may not be robust when high deformation is presented. Moreover, several assumptions such as prior knowledge about the number of existing tumours or the minimum required thickness of the slices introduces several limitations to the method. Nie *et al.* [19] used Gaussian clustering with a spatial accuracy-weighted Hidden Markov Random Fields (HMRF) that allowed them to deal with images at different resolutions without interpolation. Nowadays, advanced reconstruction techniques such as super-resolution enables to work in a high resolution voxel space by reconstructing the low resolution images. Moreover, non automated method is provided to differentiate between tumour classes and normal tissue classes after the unsupervised segmentation ends, so manual identification might be needed. Zhu *et al.* [20] developed a software based on the segmentation approach proposed by Zhang *et al.* [21], which performs an Expectation-Maximization (EM) Gaussian clustering combined whit HMRFs. Zhu *et al.* extended Zhang’s approach through a sequence of additionally morphological and thresholding operations to refine the segmentation. Such operations are not fully specified and only overall commented, so the reproducibility of their results is not possible. Vijayakumar *et al.* [22] proposed a method based on SOMs to segment tumour, necrosis, cysts, edema and normal tissues using a multi-parametric Magnetic Resonance Imaging (MRI) set. Although the learning process of SOMs is performed in an unsupervised manner, the dataset from which to infer the net structure is selected and determined manually, similar than in a supervised approach. In their work, 700 pattern observations, corresponding to 7 different tissues, where selected manually, hence converting the process in a supervised *labelling* task. Prastawa *et al.* [23] proposed a similar approach than the followed in this study. They performed an unsupervised classification based on EM algorithm and also used a brain atlas to characterize the normal tissue classes. However, they made some simplifying assumptions such as tumours should not produce too much deformation over the brain in order to allow them to use the atlas without registration. Moreover, they simplify the classification in 2 tumour classes (tumour and edema) and do not consider other tissues such as necrosis or non-enhancing tumour. Furthermore, all the unsupervised approaches proposed above applied their algorithms on its own datasets, making difficult a general comparison of the methods. Doyle *et al.* [24] also proposed an approach based on the EM clustering and a Markov Random Fields (MRF) prior. They define different penalizations between classes in the MRF, regarding to the probability of the tissues of being neighbours. However, they do not clearly specify how they relate the tissues with the classes before the unsupervised segmentation to set a different penalizations for each one.

In this work, we propose an unsupervised pipeline for GBM segmentation, able to overcome the limitations of the supervised approaches while addressing the drawbacks associated with the unsupervised learning. Our contributions concern the assessment of the performance of several unsupervised segmentation methods, including both structured and non-structured prediction algorithms, on a real public and reference dataset. We also provide a generalized method to automatically identify the classes after an unsupervised segmentation that explain the abnormal tissues in the brain. Finally, we propose a specific feature extraction and preprocessing pipeline to improve and select the relevant information of the images for the tumour segmentation task.

Our aim is to demonstrate that unsupervised segmentation algorithms can achieve competitive results, comparable to supervised approaches, but avoiding the tedious and time-consuming task of retrieving a manual labelled dataset. We evaluated our unsupervised segmentation method using the public real BRATS 2013 Leaderboard and Test sets provided for the International Image Segmentation Challenge of MICCAI Conference. The proposed method with the GMM algorithm improves the results obtained by most of the supervised approaches evaluated with the Leaderboard BRATS 2013 set, reaching the 2nd position in the rank. Our variant using the GHMRF improves the results obtained by the best unsupervised segmentation methods evaluated with the BRATS 2013 Test set, and also reaches the 7th position in the general Test rank, mainly against supervised approaches.

## Materials

In order to make our results comparable, we have used the public multi-modal BRATS dataset 2013 [25], provided for the international NCI-MICCAI 2013 Grand Challenges in Image Segmentation of MICCAI Conference. We have thoroughly evaluated our method with the Test set and the Leaderboard set, and we have made a comparison between the best algorithms that participated in the challenge and our method.

The BRATS 2013 Test set consists of multi-contrast MR scans of 10 high-grade glioma patients without the manual expert labellings. The Leaderboard set consists of 11+10 multi-contrast MR scans of high-grade glioma patients, also without the manual expert labellings. The first 11 Leaderboard patients come from to the Test set of BRATS 2012 Challenge, while the next 10 cases refer to the new Leaderboard cases for 2013 Challenge.

For each patient of the datasets, T1-weighted, T2-weighted, T2/FLAIR and post-gadolinium T1-weighted MR images were provided. All images were linearly co-registered to the post-gadolinium T1-weighted sequence, skull stripped, and interpolated to 1 mm^3^ isotropic resolution. No inter-patient registration was made to put all the images in a common reference space.

An evaluation web page was provided to upload and assess the quality of the segmentations, obtaining different metrics such as Dice, PPV, Sensitivity and Kappa indices over the different sub-compartments of the tumour such as complete tumour, enhancing tumour and core tumour.

Manual expert annotations comprise five intensity levels: Class 1) non-brain, non-tumour, necrosis, cyst and haemorrhage; class 2) surrounding edema; class 3) non-enhancing tumour; class 4) enhancing tumour core; and class 0) for everything else.

Throughout the paper, we will refer to the T1-weighted MRI sequence as *T1*, T2-weighted fast spin echo sequence as *T2*, T2/FLAIR sequence as *Flair* and post-gadolinium T1-weighted MRI sequence as *T1c*.

## Methods

### MRI preprocessing

MRI preprocessing is an active field of research that attempts to enhance and correct MR images for posterior analysis. In an unsupervised approach there is no reference or manual labelling from which to learn the models of the tissues so common artefacts such as noise or inhomogeneities may rise as erroneous classes increasing the importance of an effective preprocess. We propose the following scheme for the BRATS 2013 data: 1) Denoising, 2) Skull stripping, 3) Bias field correction and 4) Superresolution.

#### Denoising

Denoising is a standard MRI preprocessing task that aims to reduce or ideally remove the noise from an MR image. Although MRI noise has been usually modelled as a Gaussian distribution, by definition MRI noise follows a Rician distribution [26]. Diaz *et al.* [27] presented a comprehensive analysis of different denoising methods, discussing their weaknesses and strengths. Recent filters such as the Non Local Means (NLM) introduced by Buades *et al.* [28] has improved the existing techniques for MR data. Based on this approach, Manjón *et al.* [29] introduced a variant of the filter, which does not assume an uniform distribution of the noise over the image, thereby adapting the strength of the filter depending on a local estimation of the noise. The filter also deals with both correlated Gaussian and Rician noise. We used the Manjón approach to remove the noise from the BRATS images.

#### Skull stripping

**Fig 1:**
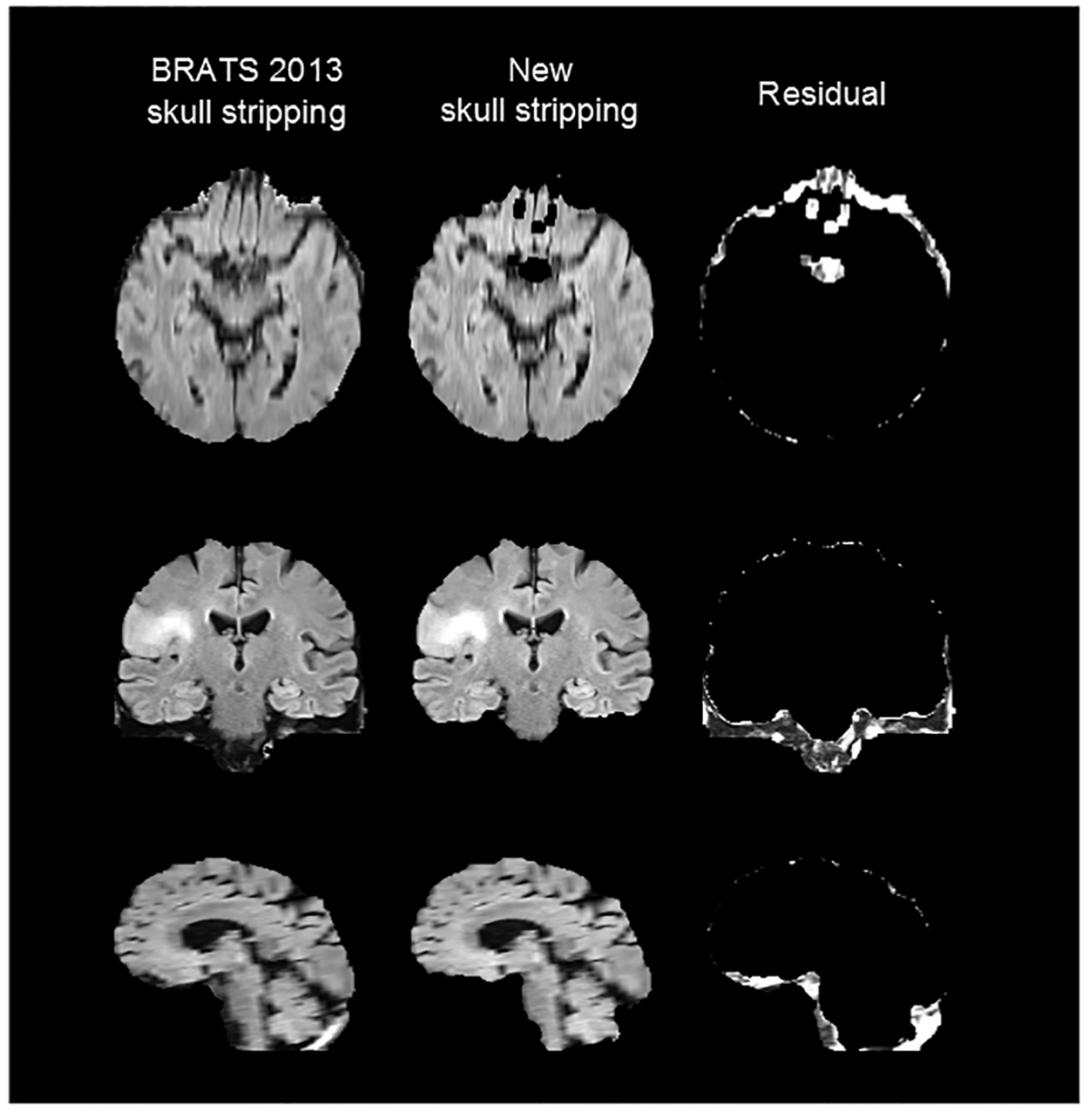
Example of new skull stripping. From left to right column: original BRATS 2013 patient image, resultant image after the new skull stripping and the remaining residual.

Skull stripping comprises the process of removing skull, extra-meningeal and non-brain tissues from the MRI sequences. Although BRATS 2013 dataset is already skull stripped, we detected several cases including partial areas of the cranium and extra-meningeal tissues. In order to improve the preprocessing of the data, we recomputed the skull stripping masks for all patients using the Brain Suite Software, and removed the non desired tissues of the MR images. Fig 1 shows an example of a patient of the BRATS 2013 dataset with the original skull stripping, the resultant image after our new skull stripping and the remaining residual.

#### Bias field correction

Intensity inhomogeneity is another common artefact present in MRI acquisitions. Magnetic field inhomogeneities are unavoidable effects consisting on low frequency signals that corrupt the images and affect their intensity levels. Typically, automated segmentation approaches are based on the assumption that the brain tissues present the same distribution of intensity among the image. Thus, intensity inhomogeneities should be corrected to ensure a correct segmentation. The popular non-parametric non-uniform intensity normalization N3 algorithm was proposed in 1998 by Sled *et al.* [30], becoming a reference technique for bias field correcting because of no tissue model was needed to perform the correction. Tustison *et al.* [31] proposed in 2010 a new implementation of N3 called N4, which improves the N3 algorithm with a better B-spline fitting function and a hierarchical optimization scheme for the bias field correction. N4 was used in our study to correct MRI inhomogeneities.

#### Super resolution

In a brain tumour lesion protocol, several MR sequences are commonly acquired normally at different resolutions, thereby introducing spatial inconsistencies when a multi-modal MR study is performed. In these cases, an upsampling or interpolation is needed to set a common voxel space for all images. Classical interpolations such as linear, cubic or splines interpolation could rise as a solution, but at the cost of having common artefacts such as partial volume effects or stair-case artefacts. In contrast, more powerful and sophisticated methods such as super resolution could improve classical interpolation by reconstructing the low resolution images recovering its high frequency components. Several super resolution schemes for MR imaging are available in the literature [32–35].

**Fig 2:**
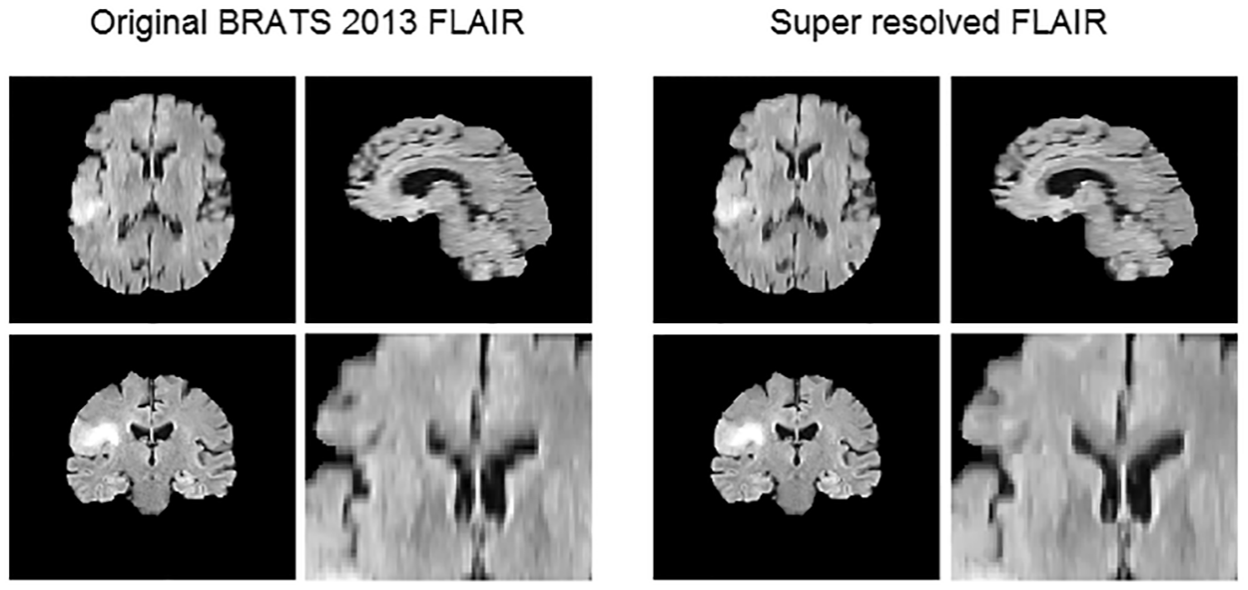
Example of super resolution using Non-local Upsampling of a Flair sequence of the BRATS 2013 dataset.

BRATS 2013 dataset comes with a 1mm^3^ isotropic voxel size resolution achieved through classic interpolation. In order to improve the resolution of these images, we employed the super resolution algorithm proposed by Manjón *et al.* [36], which exploits the self-similarity present in MR images through a patch-based non-local reconstruction process. Such method iteratively reconstructs a high resolution image by applying a Non-Local Means filter with different filter strengths, aimed to increase image regularity while constraining the image intensities to be coherent among scales through a local back-projection approach. Fig 2 shows an example of a super resolved Flair sequence of a patient of the BRATS 2013 dataset with a detailed zoom of the axial slice.

### Feature Extraction and Dimensionality Reduction

Feature extraction comprises the process of obtaining new features from the MR images to improve the discriminative power between different tissues in the brain. Although MRI intensities are the most common used features to differentiate between the brain tissues, it has been shown that including texture features in combination with MR intensities increases the performance of the segmentation algorithms [37, 38]. In this sense, we have implemented the first order statistical texture features, often called histogram derived metrics or first order central moments.

For each patient we initially obtained a derived image, named *T1d*, which consists on the absolute difference between the T1c and the T1 images [23]. This image highlights the contrast enhanced areas of the patient, such as the active tumour, helping in their discrimination. Next, for each image of the patients (T1, T1c, T2, Flair and T1d), we computed its first order texture features. Such features consist on the computation of the histograms in local 3D neighbourhoods centred at each voxel of the image, and calculate the mean, skewness and kurtosis of these histograms. We used a local 3D neighbourhood of 5 × 5 × 5 voxels.

Thus, a set *X* of 20 images is obtained for each patient, consisting on the following images:

X=(T1,T1c,T2,Flair,T1d,μT1,...,γT1,...,κT1d)

where *μ*, *γ*, and *κ* prefixes refers to the mean, skewness and kurtosis features of the corresponding images.

In order to reduce the complexity and the number of parameters to estimate to the models, a dimensionality reduction was carried out. Dimensionality reduction seeks for an efficient representation of the original high dimensional data into a lower dimensional space, but retaining or increasing its most relevant information. In our study, we used Principal Component Analysis (PCA) for dimensionality reduction. We run PCA on the *X* set and selected those principal components, whose together explained at least the 99% of the variance of the data, reducing in most cases from 20 dimensions to 5 dimensions. These images comprise the final stack of discriminant images used for the posterior segmentation.

**Fig 3:**
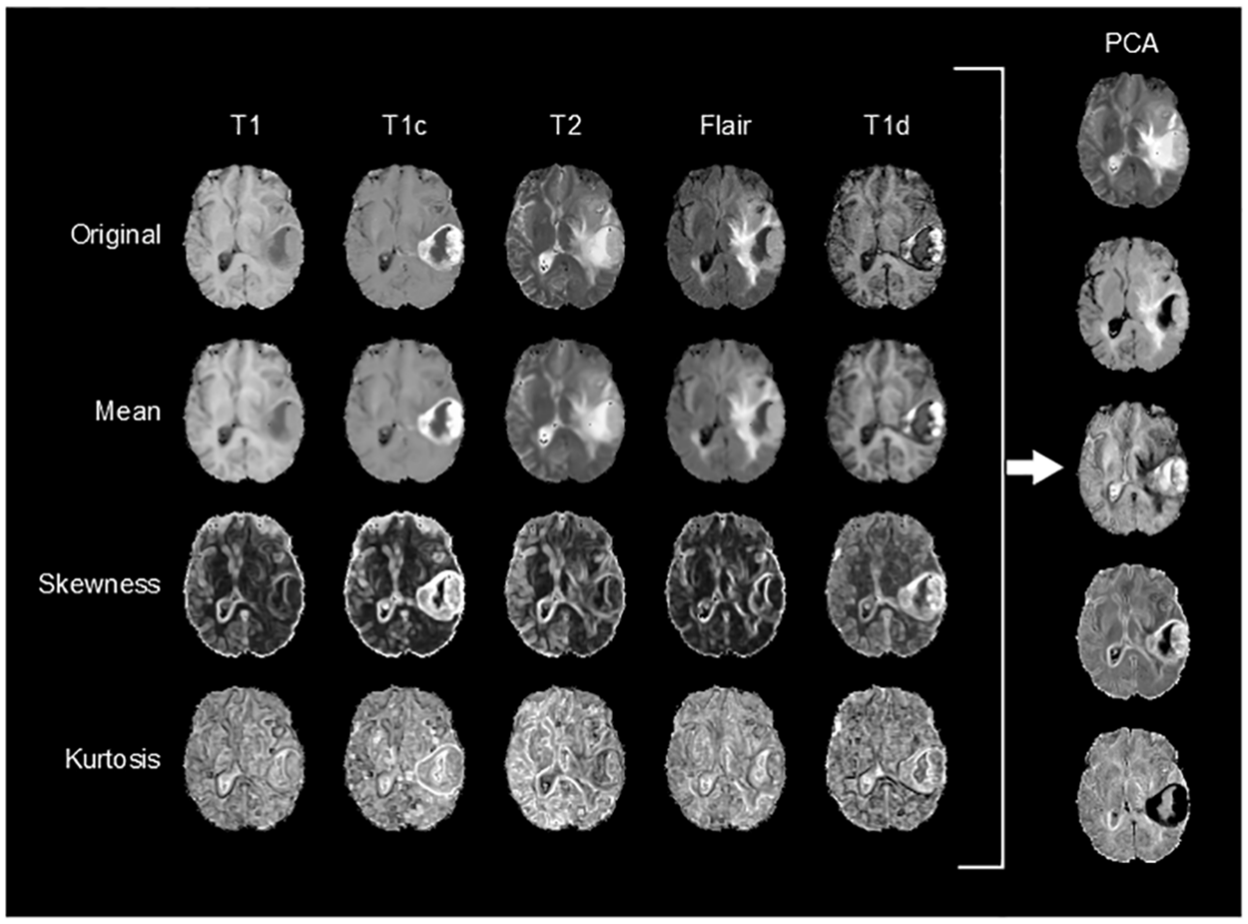
Example of feature extraction and dimensionality reduction from a patient of the BRATS 2013 dataset.

An slice example of the feature extraction and PCA dimensionality reduction process of a patient is shown in Fig 3.

### Unsupervised voxel classification

The BRATS 2013 dataset comprises 5 classes to be segmented, which in some cases a single class encloses several types of tissues (for example 0 class). This intra-class heterogeneity severely affects the performance of the unsupervised methods given that in an unsupervised segmentation scheme, the heterogeneities are usually explained by splitting the class in different clusters. Hence, in order to provide more expressibility to the unsupervised models we modelled each tissue through a mixture of 2 Gaussian distributions. Such assumption provided us a balance between the number of parameters to estimate to the models and the degrees of freedom required to explain the heterogeneity of the tissues. Therefore, we initially assumed that 7 tissues existed in the brain, which were the 1, 2, 3 and 4 classes proposed in BRATS 2013 Challenge (see Materials section), plus Gray Matter (GM), White Matter (WM) and Cerebro-Spinal Fluid (CSF), each one explained with a mixture of 2 Gaussians.

We evaluated the most popular unsupervised classification algorithms to segment the brain tissues. We divided the algorithm comparison in two groups: structured and non-structured methods. Non-structured algorithms classify data assuming an independence and identically distributed (i.i.d) condition between the voxels of the images. Structured prediction covers the range of algorithms that involve the classification of data with a specific structure, such as an image. Under the non-structured paradigm, we evaluated three methods: K-means, Fuzzy K-means and GMM clustering. In the structured prediction case we evaluated the GHMRF as the archetype of unsupervised structured learning models.

Let *X* = {**x**_1_, **x**_2_, …, **x**_*N*_} the set of voxels to be classified, where **x**_*n*_ ∈ ℝ^*D*^ represents a feature vector of *D* dimensions for voxel *n*. Let 𝓒 = {1, … *C*} the set of all possible classes for the segmentation and let *Y* = {*y*_1_, *y*_2_, …, *y*_*N*_} a segmentation of the brain volume, where *y*_*n*_ ∈ 𝓒.

#### K-means

K-means [39, 40] is an unsupervised non-structured iterative partitional clustering based on a distance minimization criterion. Its aim is to divide the data space *X* into *C* clusters, *J* = {*J*_1_, *J*_2_, …, *J*_*C*_}, so that each observation of *X* belongs to the cluster with nearest centroid. The distance criterion minimized by K-means is

$$\underset{J}{arg min}\sum_{c}^{C}\sum_{x_{n}\in J_{c}}{∥x_{n}-\mu_{c}∥}^{2}$$

From a statistical point of view, the iterative distance minimization criterion followed by K-means is equivalent to find the most likelihood parameters of a mixture of multivariate Gaussians [17], assuming a shared identity covariance matrix and uniform prior probabilities for all classes. The iterative approach followed by K-means is also demonstrated a special limit of the EM algorithm [41, 42], called *Hard-EM*, where each observation is uniquely assigned to a class with posterior probability of 1.

#### Fuzzy K-means clustering

Likewise K-means, Fuzzy K-means [43, 44] is a non-structured iterative partitional clustering that also proposes a mixture of multivariate Gaussian distribution with shared identity covariance matrix and uniform prior probabilities for all classes. However, Fuzzy K-means differs from K-means in which the assignment of an observation to a cluster is not *hard* but *fuzzy*. This means that each observation now keeps a degree of membership to each cluster (related to its posterior probability) instead of a unique assignment to a class with posterior probability of 1. In the same manner as K-means, the aim is to divide the data space *X* into *C* clusters, *J* = {*J*_1_, *J*_2_, …, *J*_*C*_}, but it also provides a vector **u**_*n*_ for each observation, which determines the membership degree of the observation *n* to the different clusters. The distance minimization criterion followed by Fuzzy K-means is

$$\underset{J}{arg min}\sum_{c}^{C}\sum_{x_{n}\in J_{c}}u_{nc}^{m}∥x_{n}-\mu_{c}{∥}^{2}\;\;\;\;\;\;\;\;\;\;1\leq m<\infty$$

where *m* controls the degree of fuzziness of the cluster *c*, typically set to 2 in absence of domain knowledge, and *u*_*nc*_ is defined as

$$u_{nc}=\frac{1}{\sum_{j}^{C}{\left(\frac{∥x_{n}-\mu_{c}{∥}^{2}}{∥x_{n}-\mu_{j}{∥}^{2}}\right)}^{\frac{2}{m-1}}}$$

where *u*_*nc*_ is proportional to the posterior probability of cluster *c* given the observation *n*.

#### GMM clustering

GMM clustering is a model-based classification algorithm whose aim is to find the maximum likelihood parameters of a Mixture of Gaussian distributions that better fit the data to be classified. GMM clustering can be seen as the generalization of K-means and Fuzzy K-means algorithms, where the hard constraints related to the shared covariance matrices and the uniform prior probabilities are relaxed. The mixture model proposed for the GMM clustering is

$$p\left(x_{n}\right)=\sum_{c}p_{c}\;𝓝\left(x_{n}|\mu_{c},\Sigma_{c}\right)$$

The EM algorithm [41] is used to find the maximum likelihood parameters of a statistical model in cases where latent variables and unknown parameters are involved, such as in the unsupervised learning paradigm. The EM algorithm starts with an initialization of the parameters $\mu_{c}^{\left(0\right)}$, $\Sigma_{c}^{\left(0\right)}$ and $p_{c}^{\left(0\right)}$ and alternates between the E step and the M step until a convergence criteria is achieved. In the E step an estimation of the posterior probability *p*(*c*|**x**_*n*_) at iteration (*k*) is computed given the current estimation of the parameters of the model. In the M step a maximum likelihood update of the parameters of the model is performed given the posterior probability computed in the E step. A convergence criteria based on the difference between the likelihood function of two consecutive iterations is usually used to ensure the convergence.

#### GHMRF

MRFs are probabilistic undirected graphical models that define a family of joint probability distributions by means of an undirected graph [45]. These graphs are used to introduce conditional dependencies between random variables of the model, which in the brain tumour segmentation task allows the model to exploit the self-similarity of the images. Such dependencies are explicitly denoted via an undirected and cyclic graph, whose vertices represent the voxels of the images and whose edges represent the dependencies between the voxels. MRFs are usually used to model the prior distribution of a probabilistic generative model, which is often expressed in terms of energy potentials. Hence, a generative model is defined as

$$p\left(X,Y\right)=p\left(X|Y\right)\;p\left(Y\right)=\frac{1}{Z}\exp(-U(X|Y)-U(Y))$$

where *Z* is called the partition function and ensures the distribution to sum 1, *U*(*Y*) is an energy function that holds the graphical model and its conditional dependencies, and *U*(*X*|*Y*) is another energy function proportional to the class-conditional *p*(*X*|*Y*) distribution of the generative model.

Nowadays, if complexity is considered, the inference algorithms for MRFs are only able to optimize undirected graphs with dependencies of order 2 (pairwise dependencies). Hence, the most widely used graphical model is the *Ising model*. The Ising model consists on a regular lattice with many vertices as voxels exist in the image, where conditional dependencies are expressed in terms of the orthogonal adjacent neighbourhood of a voxel. Therefore, we defined the *U*(*Y*) energy function as

$$\begin{array}{c} U\left(Y\right)=\sum_{(y_{n},y_{m})\in Q}\delta \left(y_{n},y_{m}\right) \end{array}$$

$$\begin{array}{c} \delta \left(y_{n},y_{m}\right)=\{\begin{array}{cc} 0, & \text{if}\;y_{n}=y_{m}\\ 1, & \text{otherwise} \end{array} \end{array}$$

where *Q* refers to the pairwise cliques of the Ising model.

Given that the *U*(*Y*) function already determines the conditional dependencies between the observations of the model, the *U*(*X*|*Y*) function can be assumed i.i.d. In our case, we modelled the *U*(*X*|*Y*) function as a Gaussian process of the form

$$\begin{array}{c} U\left(X|Y\right)=\prod_{n}U\left(x_{n}|y_{n}\right)=\prod_{n}-\log 𝓝\left(x_{n}|\mu_{c},\Sigma_{c}\right) \end{array}$$

Exact inference on the *p*(*X*, *Y*) model is intractable due to the sum over all possible configuration of labels computed in *Z*, which is a #*P*—*complete* problem. However, approximate efficient algorithms such as Iterated Conditional Modes (ICM), Monte Carlo Sampling or Graph cuts are available to compute the best labelling for pairwise MRFs. In our study we used the algorithm proposed by Komodakis *et al.* [46, 47] based on a combination of Graph cuts with primal-dual strategies.

Likewise GMM, GHMRF also finds the maximum likelihood parameters of a Mixture of Gaussian distributions that better fits the data to be classified, but imposing the structured MRF prior. A Hard-EM version of the EM algorithm is usually used to estimate the maximum likelihood parameters of the model, given that exact inference is not possible for models including MRF priors.

#### Initialization

A well-known requirement of unsupervised learning is the good initial seeding. Although the global minima is not usually reached even if a good initialization is provided, a bad initialization can lead the model to a very sub-optimal local minimum, thereby providing a poor segmentation. Several strategies such as multiple replications or intelligent initial seeding are proposed to palliate this effect. In our study, we implemented the *K-means++* algorithm proposed in [48], which provides an initialization that attempts to avoid such local minimums.

We propose the following procedure to ensure a competitive unsupervised segmentation: First, generate 100 different initializations using *K-means++* algorithm. Next, automatically select the 10 most promising initializations by minimizing the average intra-cluster sums of point-to-centroid distances of the initializations. Finally, run each unsupervised segmentation algorithm with the 10 most promising initializations and choose the best solution considering the following criteria: for K-means and Fuzzy K-means algorithms, choose again the solution with lowest intra-cluster sums of point-to-centroid distances. For GMM clustering and GHMRF, choose the solution with lowest Negative Log-Likelihood value.

### Automatic tumour classes isolation

Unsupervised segmentation produces a partitioning of the data space into several classes, each class without *semantic* sense. In other words, in the unsupervised approach, class labels do not specify a code for a specific tissue but only a mechanism to distinguish clusters different enough from each other to be considered equal. Moreover, classes between different segmentations may not always represent the same tissue, complicating its biological interpretation. Hence, automated tumour classes identification is mandatory to provide a powerful and competitive unsupervised brain tumour segmentation method. We propose the following method to automatically isolate tumour classes:

1. Identify and remove WM, GM and CSF classes
2. Remove outlier classes
3. Merge classes by statistical distribution similarities

#### Identify and remove WM, GM and CSF classes

Under the ICBM Project, an unbiased standard MR brain atlas was provided by the McConnell Brain Imaging Centre in 2009 [49, 50]. The ICBM atlas includes the T1, T2 and Proton density MR images and the WM, GM and CSF tissue probability maps. Such tissue probability maps indicate the probability for each voxel *v* of the brain to belong to a normal tissue *T* = {*WM*, *GM*, *CSF*}.

$$\begin{array}{c} \sum_{t\in T}p\left(t\;|\;v\right)=1 \end{array}$$

In our study we used the tissue probability maps to detect which classes of a segmentation explain the WM, GM and CSF tissues. However, considering that the ICBM template represents a healthy brain, we first corrected the normal tissue probability maps by removing the probability of any normal tissue *t* in the area of the tumour. Therefore, we first performed a non-linear registration of the ICBM T1 image to the patient T1 image and applied the non-linear transformation to the tissue probability maps. Following the study conducted by Klein *et al.* [51], we used the SyN algorithm [52] implemented in the Advanced Normalization Tools (ANTS) software with the cross-correlation metric to perform the non-linear registration.

**Fig 4:**
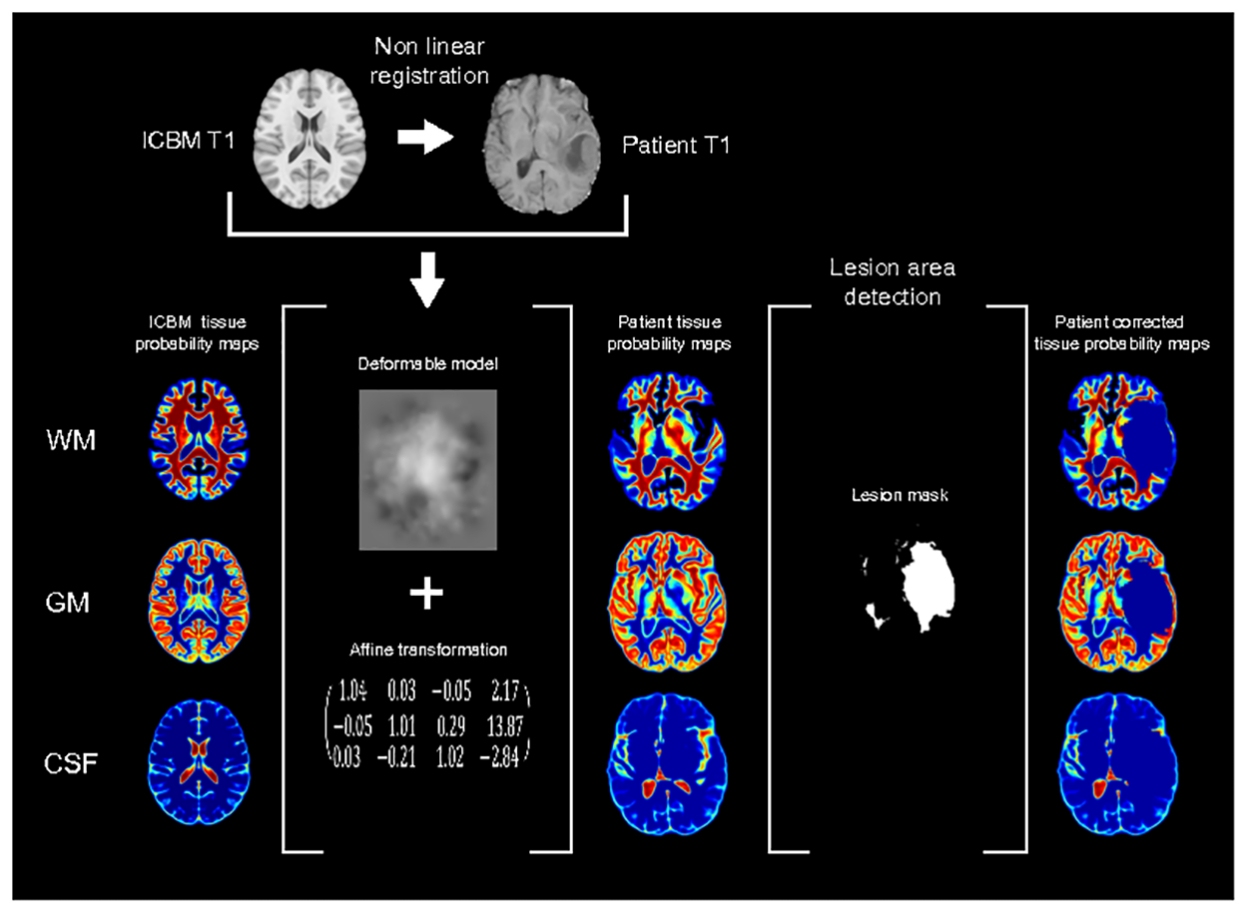
Patient tissue probability maps computation and lesion area correction.

After this step, we obtained custom normal tissue probability maps for the hypothetical healthy brain of each patient. To correct these probability maps, a roughly approximate mask of the lesion area of each patient was computed. The delineation performed by the expert radiologist of the location of the tumour is usually based on the hyper-intensity areas in the T2 and T1c sequences [2]. Following a similar criteria, we computed an approximate mask of the lesion by retrieving the Flair and T1c histograms and selecting those voxels with an intensity level higher than the median plus the standard deviation of any histogram. Next, we automatically filled the holes of the computed masks and removed the voxels that fell in the perimeter of the brain. Finally, we corrected the normal tissue probability maps of each patient by setting an *ε* probability in the area determined by their corresponding lesion mask. Fig 4 shows an example of the computation of the corrected tissue probability maps for a patient. It is worth noting that these lesion masks did not modify the shapes of the classes provided by the segmentations. Only served to approximately locate the lesion area and to correct the tissue probability maps.

Based on these custom corrected tissue probability maps, we identified which classes of a segmentation mainly explained the normal tissues *T*. Let *S* be a segmentation obtained through any unsupervised method, let *t* be a specific normal tissue, where *t* ∈ *T*, let *c* be a class of *S* and let *v* be a voxel of *S*, we computed the following probability:

$$\begin{array}{c} p\left(c|t,S\right)=\frac{\sum_{v,S(v)=c}p\left(t|v\right)}{\sum_{v}p\left(t\;|\;v\right)} \end{array}$$

Simplifying, the *p*(*c*|*t*, *S*) determines how much of the normal tissue *t* is explained by the class *c* in the segmentation *S*. Based on these percentages, we constructed two vectors, one with the *p*(*c*|*t*, *S*) values sorted in descending order, called 𝓟_*t*_, and the other with the corresponding class codes sorted in the same manner, called 𝓒_*t*_.

$$\begin{array}{c} {𝓒}_{t}=\{c\;|\;p\left(c|t,S\right)\geq p\left(c^{\prime }|t,S\right)\}\\ {𝓟}_{t}=\{p\left(c|t,S\right)\;|\;p\left(c|t,S\right)\geq p\left(c^{\prime }|t,S\right)\} \end{array}$$

Then, we computed the cumulative sum of 𝓟_*t*_, we denoted as 𝓢_*t*_, and finally choose those classes of 𝓒_*t*_ whose 𝓢_*t*_ value exceed a threshold *τ*.

$$\begin{array}{c} {𝓩}_{t}=\{{𝓒}_{t}\left(i\right)\;|\;{𝓢}_{t}\left(i\right)>\tau ,\;\;1\leq i<C\} \end{array}$$

The 𝓩_*t*_ set contains the classes of *S* that have a very low probability of explain the normal tissue *t*. Hence, we repeated the same procedure for each normal tissue *t* and computed the intersection between the 𝓩_*t*_ sets to finally isolate the classes that do not explain any normal tissue, i.e. the pathological classes

$$\begin{array}{c} 𝓩={𝓩}_{WM}\cap {𝓩}_{GM}\cap {𝓩}_{CSF} \end{array}$$

Given that our aim is to evaluate the performance of each unsupervised segmentation algorithm, all of them in the same conditions, we do not carried out a particular optimization of the *τ* threshold for each algorithm. Instead, we fixed a general threshold for all the methods to perform the tumour class identification. We choose 0.8 as a reasonably, high confidence and compatible threshold for all unsupervised methods. Note that it is not possible to fix a value of 1 due to the fact that this implies the selection of all the classes of a segmentation to explain only a single normal tissue. Moreover, the custom corrected tissue probability maps were obtained through a non-linear registration of a healthy atlas template to a pathological brain, which is not an error-free process, so the selection of the threshold should consider it.

#### Remove outlier classes

The process of identifying and removing the normal tissue classes may leave some spurious classes that should be deleted. We found that these classes frequently appear in the perimeter of the brain or in a very low rate compared to the other classes of the segmentation. The classes located at the perimeter of the brain usually correspond to the intensity gradient between the brain and the background or to the partial volume effects that the super resolution cannot remove. The low rate classes often match to outlier voxels in terms of abnormal intensity values, usually produced by artefacts in the MR acquisition.

In order to delete the perimeter unwanted classes, we first computed a binary dilated mask of the perimeter of the brain. Next, for each remaining class after the WM, GM and CSF removal, we computed their connected components and deleted those ones that fell into the mask with more than the 50% of its area. In order to remove the low rate classes we first computed the percentage of occurrence of each class of the current segmentation and deleted those ones with a value less than a 1%.

#### Merge classes by statistical distribution similarities

The heterogeneity of the tumour classes led us to assume that each tissue of the brain was modelled through at least a mixture of two Gaussians. However, the unsupervised voxel classification provided a general mixture of Gaussians over the brain, without grouping pairs of distributions. Hence, at this point, a tissue may have been represented with two or more classes of the segmentation, or by the opposite, with a single class depending on its homogeneity. Thus, it was mandatory to provide a mechanism to find class similarities that results in a homogeneous segmentation that correctly explains the final tumour tissues.

Based on the work proposed by Sáez *et al.* [53], we analysed the statistical distributions of the remaining classes after the previous steps, to find possible mixtures of classes with similar distributions. We estimated a non-parametric probability density function for each class through a kernel smoothing density estimation, and used the Jensen-Shannon divergence to measure its distances. Thus, we constructed a pairwise matrix of statistical distribution distances and used a Hierarchical Agglomerative Clustering (HAC) with an average link (Average Link (UPGMA)), to merge the similar classes.

**Fig 5:**
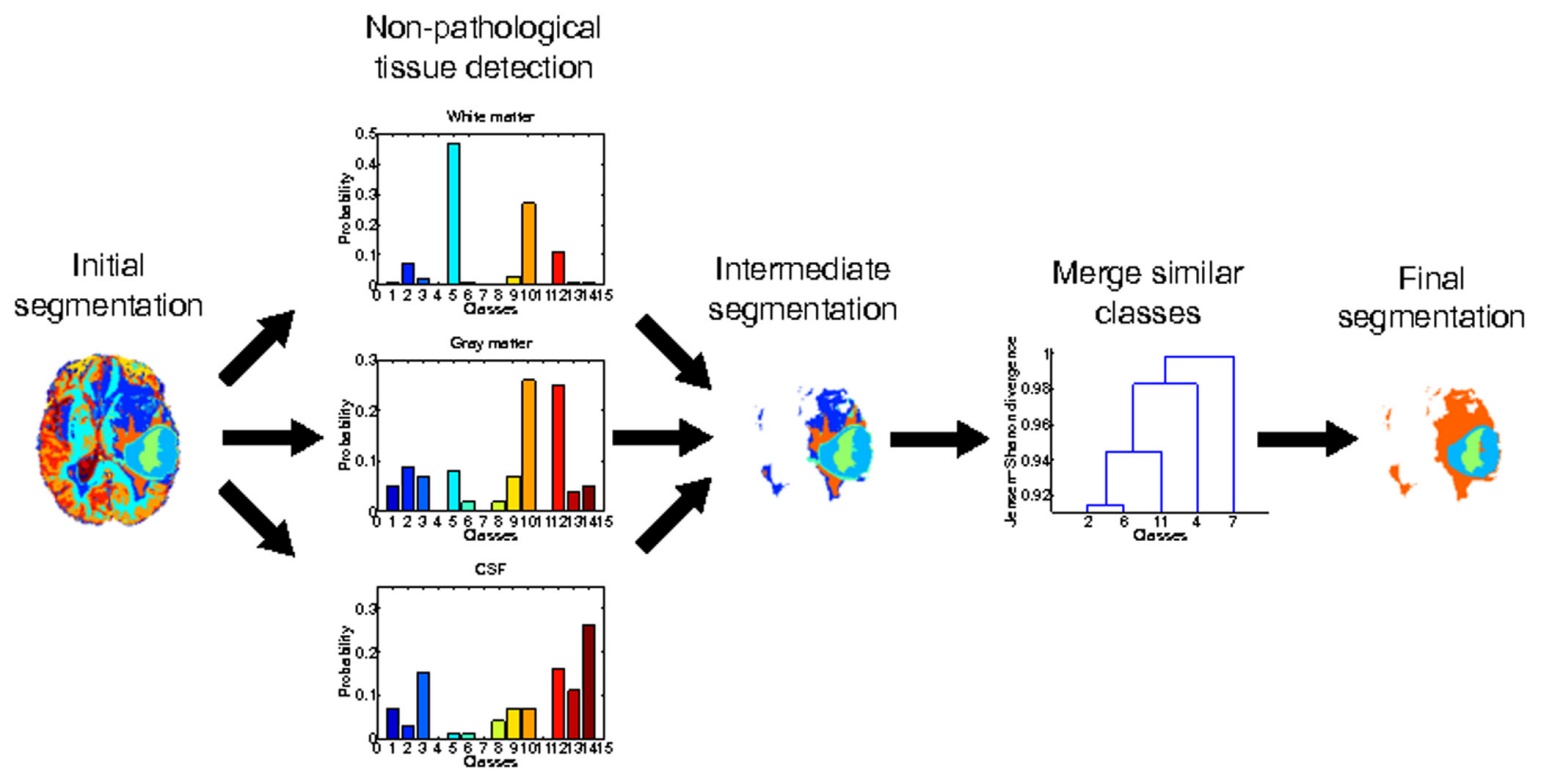
Automatic tumour class isolation process.

Due to the BRATS 2013 labelling considers 4 pathological classes to be segmented, we enforced the clustering to return a maximum of 4 classes. Note that the method is able to return less than 4 classes if the HAC finds enough similarities between the classes, however, in any other case the method is enforced to return a maximum of 4 classes. Fig 5 shows and example of the full tumour classes isolation procedure.

### Evaluation

We evaluated our unsupervised brain tumour segmentation framework with the BRATS 2013 Leaderboard and Test sets. Segmentations provided by different unsupervised methods in combination with the proposed preprocessing and postprocessing pipelines were send to the BRATS evaluation page. The figures of merit provided by the evaluation web page to assess the quality of the segmentations were:

- Dice: $\frac{2(TP+TN)}{P+N+\hat{P}+\hat{N}}$
- PPV: $\frac{TP}{TP+FP}$
- Sensitivity: $\frac{TP}{TP+FN}$
- Kappa: $\frac{P_{A}-P_{E}}{1-P_{E}}$

where *TP* refers to the true positives in the segmentation, *TN* to the true negatives, *FP* to the false positives, *FN* to the false negatives, *P* to the real positives of the ground truth, *N* to the real negatives of the ground truth, $\hat{P}$ to the estimated positives of the proposed segmentation, $\hat{N}$ to the estimated negatives of the proposed segmentation, *P*_*A*_ to the accuracy of the segmentation and *P*_*E*_ to a term that measures the probability of success by chance, defined as: $P_{E}=\left(\frac{P}{P+N}\cdot \frac{\hat{P}}{\hat{P}+\hat{N}}\right)+\left(\frac{N}{P+N}\cdot \frac{\hat{N}}{\hat{P}+\hat{N}}\right)$.

Furthermore, three different sub-compartments were evaluated for the segmentations.

1. Complete tumor: labels 1 + 2 + 3 + 4.
2. Tumor core: labels 1 + 3 + 4.
3. Enhancing tumor: label 4.

## Results

Tables 1 and 2 show the results obtained in the Test and Leaderboard sets respectively, grouped by the unsupervised algorithms tested in this study.

**Table 1:** Summary of average results obtained by the different unsupervised algorithms in combination with the proposed preprocess and postprocess over the BRATS 2013 Test set.

Classifier	Dice			PPV			Sensitiviy			Kappa
	complete	core	enhancing	complete	core	enhancing	complete	core	enhancing	
K-means	0.69	0.49	0.57	0.66	0.48	0.68	0.76	0.57	0.51	0.98
Fuzzy K-means	0.70	0.46	0.39	0.73	0.47	0.51	0.71	0.54	0.35	0.98
GMM	0.69	0.60	0.55	0.63	0.60	0.64	0.78	0.68	0.55	0.98
GHMRF	0.72	0.62	0.59	0.68	0.58	0.67	0.81	0.75	0.60	0.98

**Table 2:** Summary of average results obtained by the different unsupervised algorithms in combination with the proposed preprocess and postprocess over the BRATS 2013 Leaderboard set.

Classifier	Dice			PPV			Sensitiviy			Kappa
	complete	core	enhancing	complete	core	enhancing	complete	core	enhancing	
K-means	0.76	0.49	0.53	0.75	0.44	0.66	0.82	0.56	0.48	0.99
Fuzzy K-means	0.77	0.46	0.25	0.81	0.46	0.27	0.77	0.51	0.27	0.99
GMM	0.74	0.59	0.60	0.71	0.55	0.60	0.81	0.71	0.66	0.99
GHMRF	0.77	0.63	0.32	0.72	0.61	0.33	0.84	0.71	0.50	0.99

As it was expected, GHMRF and GMM rise as the best algorithms in combination with the proposed preprocessing and postprocessing pipelines. Almost all the metrics reveal that both algorithms obtain the best results in all the sub-compartments segmentations. Only the enhancing tumour sub-compartment in the Leaderboard set yielded low results for the GHMRF, worse than the results obtained in the other sub-compartments and datasets. Such effect is produced by the smoothing prior of the GHMRF, which is later discussed in the Discussion section.

Tables 3 and 4 show the published ranking of the BRATS competition grouped by the learning paradigm adopted by each method and the metrics and sub-compartments evaluated in the Challenge. As shown in Table 3, we achieved the 1st position in the ranking of the unsupervised methods of the Test set, and the 7th position in the general ranking, mainly against supervised approaches. Table 4 shows the Leaderboard ranking and the results achieved by our method. The proposed approach in combination with the GMM algorithm reaches the 2nd position of the Leaderboard ranking, improving the results obtained by the supervised methods, mainly in the enhancing tumour subcompartment.

**Table 3:** Ranking of the *BRATS* 2013 Test set and the position occupied by our proposed unsupervised segmentation framework with the GHMRF algorithm.

	User	Dice			PPV			Sensitiviy			Kappa
		complete	core	enhancing	complete	core	enhancing	complete	core	enhancing	
Supervised methods	Nick Tustison	0.87	0.78	0.74	0.85	0.74	0.69	0.89	0.88	0.83	0.99
	Raphael Meier	0.82	0.73	0.69	0.76	0.78	0.71	0.92	0.72	0.73	0.99
	Syed Reza	0.83	0.72	0.72	0.82	0.81	0.70	0.86	0.69	0.76	0.99
	Liang Zhao	0.84	0.70	0.65	0.80	0.67	0.65	0.89	0.79	0.70	0.99
	Nicolas Cordier	0.84	0.68	0.65	0.88	0.63	0.68	0.81	0.82	0.66	0.99
	Joana Festa	0.72	0.66	0.67	0.77	0.77	0.70	0.72	0.60	0.70	0.98
Unsupervised methods	**This work. GHMRF**	**0.72**	**0.62**	**0.59**	**0.68**	**0.58**	**0.67**	**0.81**	**0.75**	**0.60**	**0.98**
	Senan Doyle	0.71	0.46	0.52	0.66	0.38	0.58	0.87	0.70	0.55	0.98

**Table 4:** Ranking of the *BRATS* 2013 Leaderboard set and the position occupied by our proposed unsupervised segmentation framework with the GMM algorithm.

	User	Dice			PPV			Sensitiviy			Kappa
		complete	core	enhancing	complete	core	enhancing	complete	core	enhancing	
Supervised method	Nick Tustison	0.79	0.65	0.53	0.83	0.70	0.51	0.81	0.73	0.66	0.99
Unsupervised method	**This work. GMM**	**0.74**	**0.59**	**0.60**	**0.71**	**0.55**	**0.60**	**0.81**	**0.71**	**0.66**	**0.99**
Supervised methods	Liang Zhao	0.79	0.59	0.47	0.77	0.55	0.50	0.85	0.77	0.53	0.99
	Raphael Meier	0.72	0.60	0.53	0.65	0.62	0.48	0.88	0.69	0.64	0.99
	Syed Reza	0.73	0.56	0.51	0.68	0.64	0.48	0.79	0.57	0.63	0.99
	Nicolas Cordier	0.75	0.61	0.46	0.79	0.61	0.43	0.78	0.72	0.52	1.00

Table 5 shows the average time in minutes required to obtain a segmentation for a single patient, including the preprocessing and postprocessing of the data. Segmentations were computed in an Intel Xeon E5-2620 with 64GB of RAM using multi-threading. The preprocessing time includes the denoising, bias field correction, skull-stripping and super resolution steps. The unsupervised classification time involves the parallel computation of the 10 different segmentations starting from the K-means++ initialization, and the posterior selection of the best solution. As expected, the more complex and sophisticated the algorithms are, the longer they take to reach the solution. The postprocessing time refers to the automated tumour class isolation step, the outlier class removal and the merging process of similar statistical distribution classes. Such process includes the non-linear registration of the ICBM template to the patient T1 image, which practically covers the entire time of the postprocessing stage. It is worth noting that the non-linear ICBM registration is performed only once for all the unsupervised segmentation algorithms.

**Table 5:** Average computational times in minutes for the whole segmentation pipeline for a single patient.

Algorithm	Preprocess	Unsupervised classification	Postprocess	Total
K-means	13 ± 3	9 ± 5	88 ± 19	110 ± 27
Fuzzy K-means		29 ± 3		130 ± 25
GMM		41 ± 7		142 ± 29
GHMRF		39 ± 10		140 ± 32

**Fig 6:**
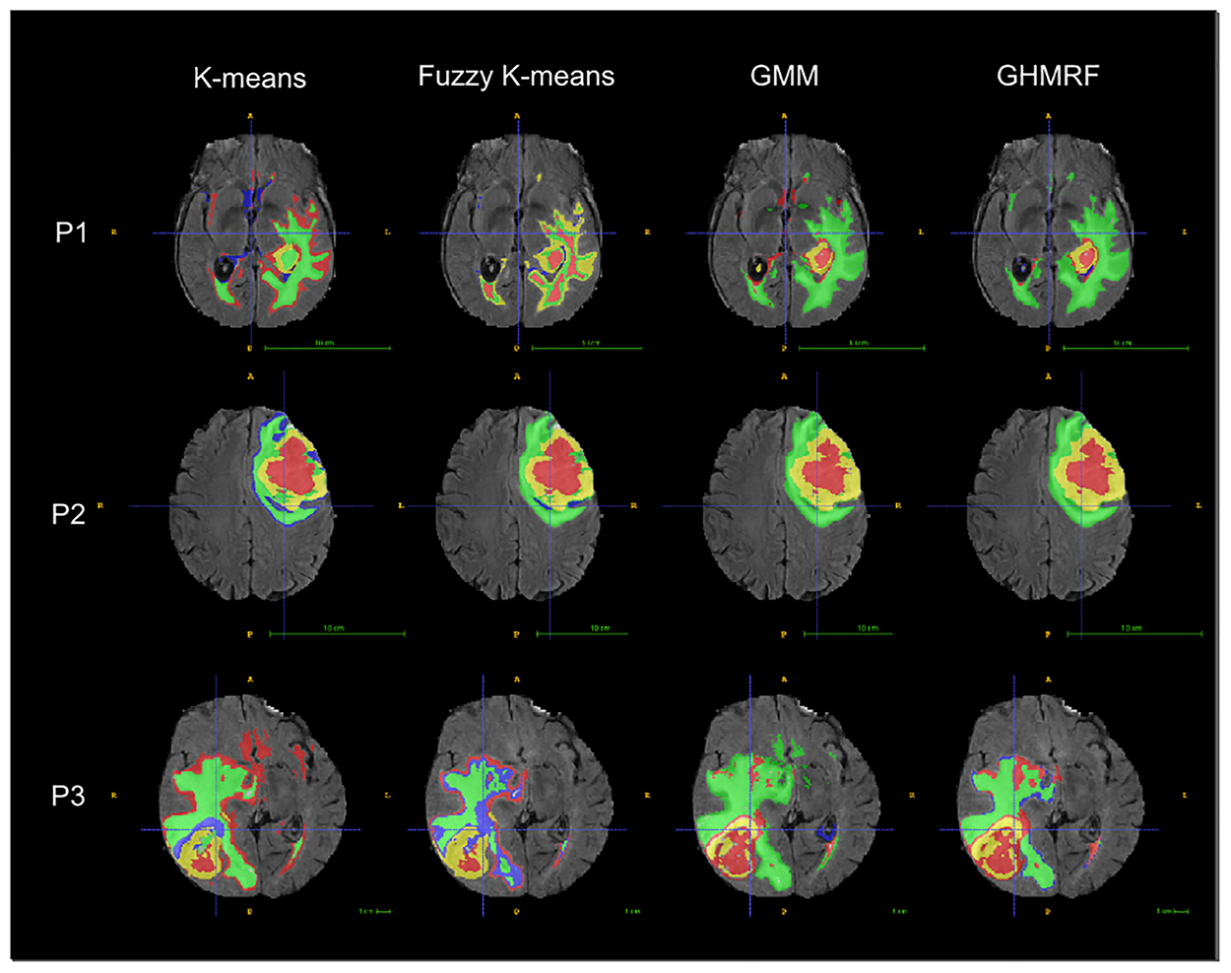
Examples of final segmentations of 3 patients of BRATS 2013 dataset computed by the different unsupervised algorithms.

Finally, examples of segmentations achieved by different unsupervised segmentation algorithms in our system are showed in Fig 6.

## Discussion

The proposed unsupervised brain tumour segmentation method is confirmed as a viable alternative for GBM segmentation, as it has demonstrated to achieve competitive results in a public real reference dataset for brain tumour segmentation. The method improves the results obtained by the other unsupervised segmentation approaches evaluated in the BRATS 2013 Challenge, and obtains competitive results with respect to supervised methods, without requiring a manual expert labelling.

The proposed unsupervised segmentation method comprises four stages: MRI preprocessing, feature extraction and dimensionality reduction, unsupervised voxel classification and automatic tumour classes isolation. Concerning the preprocessing stage, consolidated state of the art techniques that provide efficient solutions to enhance the information of the MR images were employed. However, some preprocessing techniques are primarily oriented to non-pathological brains. This is the case of bias field correction. In our experiments, we found that the estimation of the magnetic field inhomogeneities with the N4 algorithm presented problems primarily with Flair sequences. The hyper-intensity presented in the Flair sequence by the edema was confused frequently with inhomogeneities of the magnetic field, thereby reducing its intensity. In order to overcome this problem we reduced the number of iterations of the algorithm to remove as much inhomogeneities as possible, while keeping the intensities of the lesion. Such solution assumes a non optimal removal of the magnetic field inhomogeneities, but allows to save the information contained in the lesion area, which becomes more important to the segmentation task. We empirically set a maximum of 10 iterations at each scale of the multi-scale approach of the N4 algorithm.

Several unsupervised classification algorithms were evaluated to assess its pros and cons, ranging from the most restrictive algorithms in terms of class-conditional probabilistic models (K-means and Fuzzy K-means) to more sophisticated models with more degrees of freedom such as GMM or GHMRF. The last one, also introduces statistical dependencies between adjacent variables of the model, that penalizes neighbouring voxels with different classes. Hence, this structured prior aims to model the self similarity presented in the images, leading the algorithm to a more homogeneous segmentation than the non-structured classification techniques.

Therefore, it was expected that the less restrictive algorithms in terms of class-conditional probabilistic model were likely to achieve better results based on the hypothesis that these algorithms learn a more flexible model that better fits the data to be classified. Moreover, structured algorithms are also expected to obtain better results based on the hypothesis that these algorithms introduce mechanisms to model the self similarity of the images through the conditional dependencies defined for the data. Tables 1 and 2 confirms such hypotheses. Both GMM and GHMRF rise as the best algorithm tested in almost all the metrics returned by the evaluation web page. Only the results obtained by the GHMRF model in the enhancing sub-compartment of the Leaderboard set were not comparable with the other sub-compartments and datasets results. This effect is produced due to the smoothing prior imposed by the GHMRF, which is too strong in some cases. We revised the cases that achieved low results in the *enhancing tumour* sub-compartment and realized that most of them had a large necrotic core and a thin low-brightness enhancing tumour ring. We also revised the K-means++ initializations and realized that the enhancing tumour was partially segmented in some cases but finally lost in the final segmentation due to the hard smoothing prior in the necrotic class. We are now working in the introduction of different penalizations for the classes, depending on their statistical distribution similarities to avoid this over-smoothing.

It is worth noting that we obtained better results on the Leaderboard set (Table 4) than in the Test set (Table 3), in contrast with the rest of participants. This effect may have been produced by the fact that the Leaderboard set may include more heterogeneities and differences with respect to the Training set than the Test set, thereby directly affecting the supervised approaches performance. Unsupervised paradigm avoids this possible overfitting by building a particular model for each patient considering only its own data, therefore achieving better results in the Leaderboard set against most of the supervised approaches evaluated.

In future work, we plan to improve our feature extraction process by analysing the influence of the texture images in the final segmentations and including more sophisticated textures such as the Haralick texture features. Furthermore, we plan to extend our unsupervised methodology to the analysis and segmentation of Perfusion Weighted Images (PWI) in combination with anatomical images. The biomarkers obtained from PWI might discover relevant segmentations by adding additional valuable functional information about the tissues. We consider that research efforts should be aligned with quantitative MRI by providing powerful systems that leverage the information contained in these images.

## References

[1] Wen PY, Macdonald DR, Reardon DA, Cloughesy TF, Sorensen AG, Galanis E, et al Updated response assessment criteria for high-grade gliomas: Response assessment in neuro-oncology working group. Journal of Clinical Oncology. 2010;28: 1963–1972. 10.1200/JCO.2009.26.3541 20231676

[2] Bauer S, Wiest R, Nolte LP, Reyes M. A survey of MRI-based medical image analysis for brain tumor studies. Physics in Medicine and Biology. 2013;58: R97–129. 10.1088/0031-9155/58/13/R97 23743802

[3] Dolecek TA, Propp JM, Stroup NE, Kruchko C. Cbtrus statistical report: Primary brain and central nervous system tumors diagnosed in the united states in 2005–2009. Neuro-Oncology. 2012;14: 1–49. 10.1093/neuonc/nos218 PMC3480240 23095881

[4] von Deimling A. Gliomas (Recent Results in Cancer Research). Heidelberg: Springer; 2009.

[5] Gordillo N, Montseny E, Sobrevilla P. State of the art survey on MRI brain tumor segmentation. Magnetic Resonance Imaging. 2013;31: 1426–1438. 10.1016/j.mri.2013.05.002 23790354

[6] Verma R, Zacharaki EI, Ou Y, Cai H, Chawla S, Lee SK, et al Multiparametric tissue characterization of brain neoplasms and their recurrence using pattern classification of MR images. Academic Radiology. 2008;15: 966–977. 10.1016/j.acra.2008.01.029 18620117 PMC2596598

[7] Ruan S, Zhang N, Liao Q, Zhu Y. Image fusion for following-up brain tumor evolution. IEEE International Symposium on Biomedical Imaging: From Nano to Macro. 2011;1: 281–284.

[8] Jensen TR, Schmainda KM. Computer-aided detection of brain tumor invasion using multiparametric MRI. Journal of Magnetic Resonance Imaging. 2009;30: 481–489. 10.1002/jmri.21878 19711398 PMC4321878

[9] Lee CH, Wang S, Murtha A, Brown M, Greiner R. Segmenting brain tumors using pseudo–conditional random fields. Medical Image Computing and Computer-Assisted Intervention. 2008;11: 359–366. 18979767

[10] Bauer S, Nolte LP, Reyes M. Fully automatic segmentation of brain tumor images using support vector machine classification in combination with hierarchical conditional random field regularization. Medical Image Computing and Computer-Assisted Intervention. 2011;14: 354–361. 22003719 10.1007/978-3-642-23626-6_44

[11] Breiman L. Random forests. Machine Learning 2001;45: 5–32. 10.1023/A:1010933404324

[12] Meier R, Bauer S, Slotboom J, Wiest R, Reyes M. A hybrid model for multimodal brain tumor segmentation. Proceedings of NCI-MICCAI BRATS. 2013;1: 31–37.

[13] Festa J, Pereira S, Mariz JA, Sousa N, Silva CA. Automatic brain tumor segmentation of multi-sequence mr images using random decision forests. Proceedings of NCI-MICCAI BRATS. 2013;1: 23–26.

[14] Reza S, Iftekharuddin K. Multi-class abnormal brain tissue segmentation using texture features. Proceedings of NCI-MICCAI BRATS. 2013;1: 38–42.

[15] Tustison N, Wintermark M, Durst C, Avants B. ANTs and árboles. Proceedings of NCI-MICCAI BRATS. 2013;1: 47–50.

[16] Wagstaff KL. Intelligent Clustering with instance-level constraints. PhD Thesis, Cornell University. 2002.

[17] Duda R, Hart P, Stork D. Pattern classification. 2nd ed. New York: Wiley-Interscience; 2000.

[18] Fletcher-Heath LM, Hall LO, Goldgof DB, Murtagh F. Automatic segmentation of non-enhancing brain tumors in magnetic resonance images. Artificial Intelligence in Medicine. 2001;21: 43–63. 10.1016/S0933-3657(00)00073-7 11154873

[19] Nie J, Xue Z, Liu T, Young GS, Setayesh K, Guo L, et al Automated brain tumor segmentation using spatial accuracy-weighted hidden Markov random field. Computerized Medical Imaging and Graphics. 2009;33: 431–441. 10.1016/j.compmedimag.2009.04.006 19446435 PMC2739047

[20] Zhu Y, Young GS, Xue Z, Huang RY, You H, Setayesh K, et al Semi-automatic segmentation software for quantitative clinical brain glioblastoma evaluation. Academic Radiology. 2012;19: 977–985. 10.1016/j.acra.2012.03.026 22591720 PMC3515056

[21] Zhang Y, Brady M, Smith S. Segmentation of brain MR images through a hidden Markov random field model and the expectation-maximization algorithm. IEEE Transactions on Medical Imaging. 2001;20: 45–57. 10.1109/42.906424 11293691

[22] Vijayakumar C, Damayanti G, Pant R, Sreedhar C. Segmentation and grading of brain tumors on apparent diffusion coefficient images using self-organizing maps. Computerized Medical Imaging and Graphics. 2007;31: 473–484. 10.1016/j.compmedimag.2007.04.004 17572068

[23] Prastawa M, Bullit E, Moon N, Leemput K, Gerig G. Automatic brain tumor segmentation by subject specific modification of atlas priors. Academic Radiology. 2003;10: 1341–1348. 10.1016/S1076-6332(03)00506-3 14697002 PMC2430604

[24] Doyle S, Vasseur F, Dojat M, Forbes F. Fully automatic brain tumor segmentation from multiple MR sequences using hidden Markov fields and variational EM. Proceedings of NCI-MICCAI BRATS. 2013;1: 18–22.

[25] Menze Bjoern, Jakab Andras, Bauer Stefan, Kalpathy-Cramer Jayashree, Farahani Keyvan, Kirby Justin, et al The Multimodal Brain Tumor Image Segmentation Benchmark (BRATS). IEEE Transactions on Medical Imaging. 2014; 1–33. 10.1109/TMI.2014.2377694 25494501 PMC4833122

[26] Gudbjartsson H, Patz S. The rician distribution of noisy MRI data. Magnetic Resonance in Medicine. 1995;34: 910–914. 10.1002/mrm.1910340618 8598820 PMC2254141

[27] Diaz I, Boulanger P, Greiner R, Murtha A. A critical review of the effects of de-noising algorithms on MRI brain tumor segmentation. Proceedings of Engineering in Medicine and Biology Society. 2011;1: 3934–3937. 10.1109/IEMBS.2011.6090977 22255200

[28] Buades A, Coll B, Morel JM. A review of image denoising algorithms, with a new one. Multiscale Model & Simulation. 2005;4: 490–530. 10.1137/040616024

[29] Manjón JV, Coupé P, Martí-Bonmatí L, Collins DL, Robles M. Adaptive non-local means denoising of MR images with spatially varying noise levels. Journal of Magnetic Resonance Imaging. 2010;31: 192–203. 10.1002/jmri.22003 20027588

[30] Sled J, Zijdenbos A, Evans A. A nonparametric method for automatic correction of intensity nonuniformity in MRI data. IEEE Transactions on Medical Imaging. 1998;17: 87–97. 10.1109/42.668698 9617910

[31] Tustison N, Avants B, Cook P, Zheng Y, Egan A, Yushkevich PA, et al N4ITK: Improved N3 bias correction. IEEE Transactions on Medical Imaging. 2010;29: 1310–1320. 10.1109/TMI.2010.2046908 20378467 PMC3071855

[32] Plenge E, Poot D, Niessen W, Meijering E. Super-resolution reconstruction using cross-scale self-similarity in multi-slice MRI. Medical Image Computing and Computer-Assisted Intervention. 2013;16: 123–130. 24505752 10.1007/978-3-642-40760-4_16

[33] Manjón JV, Coupé P, Buades A, Collins DL, Robles M. MRI superresolution using self similarity and image priors. International Journal of Biomedical Imaging. 2010;17: 1–12. 10.1155/2010/425891 PMC3004412 21197094

[34] Rousseau F. A non-local approach for image super-resolution using intermodality priors. Medical Image Analysis. 2010;14: 594–605. 10.1016/j.media.2010.04.005 20580893 PMC2947386

[35] Protter M, Elad M, Takeda H, Milanfar P. Generalizing the Non Local-Means to super-resolution reconstruction. IEEE Transactions on Image Processing. 2009;18: 36–51. 10.1109/TIP.2008.2008067 19095517

[36] Manjón JV, Coupé P, Buades A, Fonov V, Collins DL, Robles M. Non-local MRI upsampling. Medical Image Analysis. 2010;14: 784–792. 10.1016/j.media.2010.05.010 20566298

[37] Kassner A, Thornhill R. Texture analysis: A review of neurologic MR imaging applications. American Journal of Neuroradiology. 2010;31: 809–816. 10.3174/ajnr.A2061 20395383 PMC7964174

[38] Ahmed S, Iftekharuddin KM, Vossough A. Efficacy of texture, shape, and intensity feature fusion for posterior-fossa tumor segmentation in MRI. IEE Transaction on Information Technology in Biomedicine. 2011;15: 206–213. 10.1109/TITB.2011.2104376 21216716

[39] Lloyd S. Least squares quantization in PCM. IEEE Transactions on Information Theory. 1982;28: 129–137. 10.1109/TIT.1982.1056489

[40] MacQueen J. Some methods for classification and analysis of multivariate observations. Proceedings of the Fifth Berkeley Symposium on Mathematical Statistics and Probability. 1967;1: 281–297.

[41] Dempster AP, Laird NM, Rubin DB. Maximum Likelihood from Incomplete Data via the EM Algorithm. Journal of the Royal Statistical Society. 1977;39: 1–38.

[42] Bishop C. Neural Networks for Pattern Recognition. New York: Oxford University Press, Inc; 1995.

[43] Dunn JC. A fuzzy relative of the isodata process and its use in detecting compact well-separated clusters. Journal of Cybernetics. 1973;3: 32–57. 10.1080/01969727308546046

[44] Bezdek JC. Pattern Recognition with Fuzzy Objective Function Algorithms. Norwell: Kluwer Academic Publishers; 1981.

[45] Hammersley, JM, Clifford, P. Markov fields on finite graphs and lattices. 1971.

[46] Komodakis N, Tziritas G. Approximate labeling via graph cuts based on linear programming. IEEE Transactions on Pattern Analysis and Machine Intelligence. 2007;29: 1436–1453. 10.1109/TPAMI.2007.1061 17568146

[47] Komodakis N, Tziritas G, Paragios N. Performance vs computational efficiency for optimizing single and dynamic MRFs: Setting the state of the art with primal-dual strategies. Computer Vision and Image Understanding. 2008;112: 14–29. 10.1016/j.cviu.2008.06.007

[48] Arthur D, Vassilvitskii S. K-means++: The advantages of careful seeding. Proceedings of the Eighteenth Annual ACM-SIAM Symposium on Discrete Algorithms. 2007;1: 1027–1035.

[49] Fonov VS, Evans AC, Botteron K, Almli CR, McKinstry RC, Collins DL, et al Unbiased average age-appropriate atlases for pediatric studies. NeuroImage. 2011;54: 313–327 10.1016/j.neuroimage.2010.07.033 20656036 PMC2962759

[50] Fonov VS, Evans AC, McKinstry RC, Almli CR, Collins DL. Unbiased nonlinear average age-appropriate brain templates from birth to adulthood. NeuroImage. 2009;47: S102 10.1016/S1053-8119(09)70884-5

[51] Klein A, Andersson J, Ardekani BA, Ashburner J, Avants B, Chiang MC, et al Evaluation of 14 nonlinear deformation algorithms applied to human brain MRI registration. NeuroImage. 2009;46: 786–802. 10.1016/j.neuroimage.2008.12.037 19195496 PMC2747506

[52] Avants B, Epstein C, Grossman M, Gee J. Symmetric diffeomorphic image registration with cross-correlation: Evaluating automated labeling of elderly and neurodegenerative brain. Medical Image Analysis. 2008;12: 26–41. 10.1016/j.media.2007.06.004 17659998 PMC2276735

[53] Saez C, Robles M, Garcia-Gomez JM. Stability metrics for multi-source biomedical data based on simplicial projections from probability distribution distances. Statistical Methods in Medical Research 2014; In press. 10.1177/0962280214545122 25091808

